# Mechanism-Based Allylic Carbasugar Chlorides That Form Covalent Intermediates with α- and β-Galactosidases

**DOI:** 10.3390/molecules29204870

**Published:** 2024-10-14

**Authors:** Oluwafemi Akintola, Sandeep Bhosale, Andrew J. Bennet

**Affiliations:** Department of Chemistry, Simon Fraser University, Burnaby, BC V5A 1S6, Canada; akintolaoluwafemi@rocketmail.com

**Keywords:** mechanism-based, glycoside hydrolase, covalent inhibitor, carbasugar, selectivity, galactosidase

## Abstract

Glycoside hydrolases have been implicated in a wide range of human conditions including lysosomal storage diseases. Consequently, many researchers have directed their efforts towards identifying new classes of glycoside hydrolase inhibitors, both synthetic and from natural sources. A large percentage of such inhibitors are reversible competitive inhibitors that bind in the active site often due to them possessing structural features, often a protonatable basic nitrogen atom, that mimic the enzymatic transition state. We report that mechanism-based small molecule *galacto*-like configured cyclohexenyl carbasugars form reversible covalent complexes with both α-galactosidase and β-galactosidase. In addition, we show that the β-galactosidase from *Aspergillus oryzae* reacts with three different carbasugar inhibitors, with three different second-order rate constants (*k*_inact_/*K*_i_), to give the same enzyme–carbasugar covalent intermediate. The surprising observation that the α-galacto-configured inhibitor covalently labels the *A. oryzae* β-galactosidase highlights the catalytic versatility of glycoside hydrolases. We expect that cyclohexenyl covalent inhibitors will become an important class of compounds in the chemical biologist’s tool box.

## 1. Introduction

There are three main types of polymeric building blocks found in nature, and these are DNA/RNA, proteins, and polysaccharides. Of note, it is carbohydrates that are the most structurally diverse and as these sugars are essential components for all living systems, their metabolism is central to life, with their addition or removal being catalyzed by enzymes that are found in all kingdoms of life [1]. The two main biological catalysts for the addition and removal of sugar units are called glycosyltransferases (GTs) [2] and glycoside hydrolases (GHs or glycosidases), respectively [3,4]. The stereochemical outcome at the anomeric center for the glycoside hydrolases that catalyze nucleophilic substitution reactions at this carbon atom occur with either retention (the same stereochemistry as the substrate; retaining GHs) or inversion (inverting GHs) [5,6]. Bioinformatics has enabled classification based on amino acid sequence similarities of these hydrolases into over 180 GH families [7,8].

As a consequence of the growing recognition of the importance of GHs in numerous diverse biological processes and a renewed interest in biologically active covalent inhibitors [9,10], a burgeoning interest exists for the development of chemical probes that can modulate GH activities, including small molecule mechanism-based inhibitors [11,12,13]. These classes of inhibitors incorporate structural elements, and the appropriate stereochemistry of hydroxyl substituents so as to take advantage of a retaining GH’s mechanism of action by the formation of either a temporary or permanent covalently bound enzyme intermediate. Indeed, carbasugar covalent inhibitors have been reported, which mimic glycosidic substrates by replacing the C5 carbon and the endocyclic oxygen with a variety of functional groups, cyclopropyl rings [14,15], alkenes, [16,17,18], epoxides [19,20], or aziridines [21,22].

**Figure 1:**
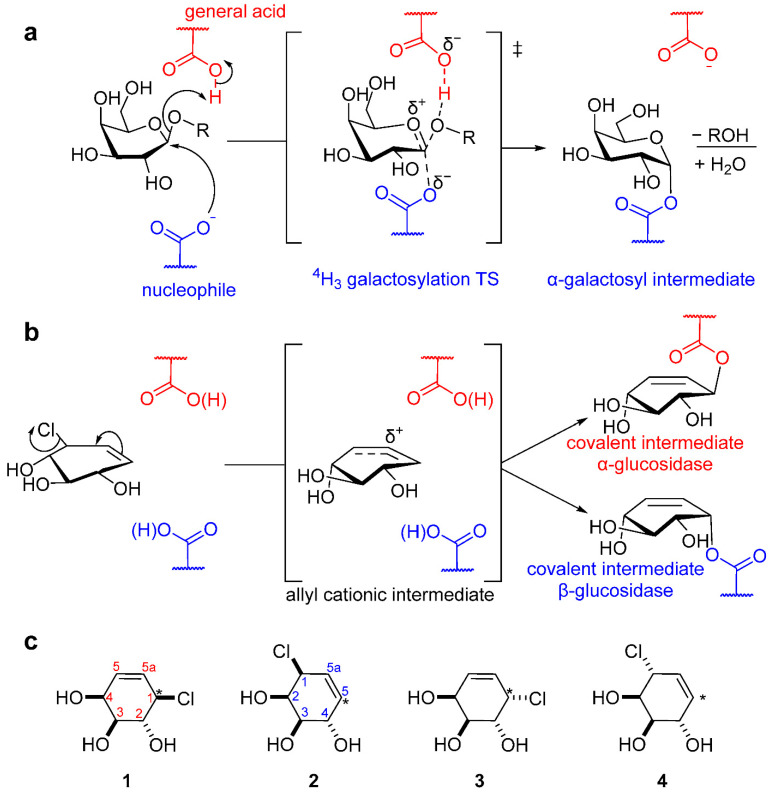
The mechanism of action for substrate hydrolysis, covalent inhibition, and structures of covalent inhibitors in the current study. (**a**) The currently accepted mechanism for the formation of a galactosyl-enzyme covalent intermediate for β-galactosidases. (**b**) The proposed mechanism for the formation of an enzyme-bound *gluco*-like allylic cation that is trapped by the enzymatic nucleophile. Nucleophiles are color coded: red for α-glucosidases and blue for β-glucosidases. (**c**) Structures of covalent inhibitors made for the current study; the pseudoanomeric carbon is labeled with an asterisk for all structures. The carbon framework is numbered using carbohydrate nomenclature, red for inhibitors with a pseudoanomeric leaving group and blue for a C5 leaving group.

Recently, we have shown that carbasugars lacking the C6 hydroxymethyl (CH_2_OH), which possess C2–C4 *xylo*-configured hydroxyl groups, form covalent intermediates with glucocerebrosidase (GCase), which is a retaining GH from family 30, and yeast α-glucosidase (GH13). Of particular note, we reported that an allylic carbasugar chloride in which the leaving group is located at C5 of the ring covalently label both an α-glucosidase and a β-glucosidase [23]. The proposed mechanism by which this novel carbasugar covalently labels anomerically distinct GHs is shown in Figure 1b. Herein, we report an extension of this strategy to target both an α-galactosidase (GH27) and a β-galactosidase (GH35), which operates by the mechanism shown in Figure 1a, by synthesizing and testing the inhibitory activities of four cyclohexenyl-based carbasugar chlorides **1**–**4** (Figure 1c).

## 2. Results and Discussion

### 2.1. Synthesis of Galacto-Configured Allylic Carbasugars

**Scheme 1:**
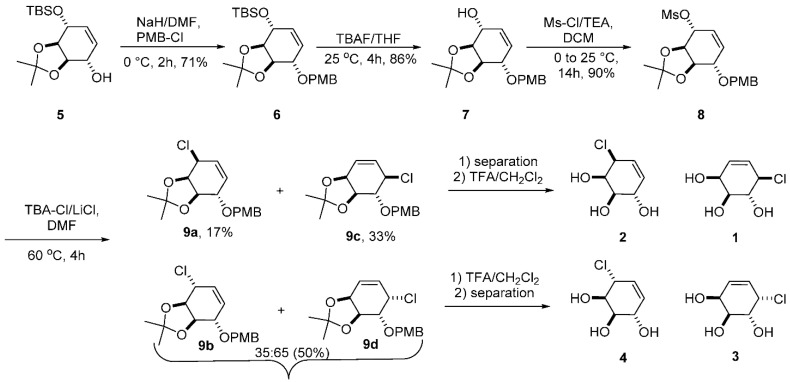
Synthesis of potential α- and β-galactosidase covalent inhibitors **1**–**4**.

Our synthetic route (Scheme 1) to the potential covalent inhibitors **1**–**4** started with d-(–)-quinic acid and took advantage of its *cis*-hydroxyl groups to make the key acetonide intermediate **5** in eight steps following literature procedures [24,25]. Following *p-*methoxybenzyl protection, we removed the silyl-protecting group to give intermediate **7**. The mesylation of the allylic hydroxyl group gave **8**, a compound that is set up for the simultaneous formation of all four required carbasugar chlorides in protected form (**9a**–**9d**) in a single reaction. That is, an S_N_1 ionization of the mesylate gives an allylic cation intermediate that can be trapped to give inversion and retention at the departing center (**9a** and **9b**) as well as the two rearranged products (**9c** and **9d**) by the attack of chloride on the remote allylic center. We successfully separated **9a** and **9c** by column chromatography, but we also obtained an inseparable mixture of **9b** and **9d** (ratio 35:65). The two pure diastereomers (**9a** and **9c**) were independently deprotected under acidic conditions using TFA to afford pure carbasugar chloride **2** (75%) and **1** (85%), respectively. We similarly deprotected the mixture of **9b** and **9d** after separation **4** and **3**, respectively.

### 2.2. Kinetics of Galactosidase Covalent Inhibition and Reactivation

**Figure 2:**
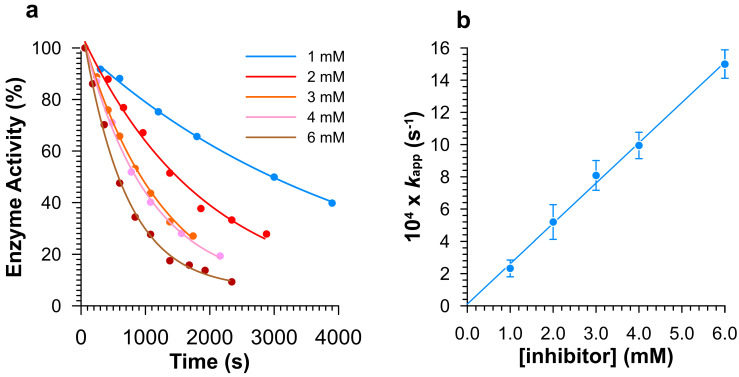
Plots for the loss of enzymatic activity for the human GH27 α-galactosidase that result from incubation with **2**. (**a**) The observed loss of enzyme activity that results from different concentrations of **2** [1 mM: blue; 2 mM red; 3 mM orange; 4 mM pink; 6 mM brown]; all lines are the least-squares fit to a standard first-order exponential decay. (**b**) The linear plot of the apparent rate constant for inactivation of Human α-galactosidase caused by incubation with **2**. All experiments were run in a 50 mM sodium citrate buffer, pH 4.6, containing 50 mM NaCl and 0.1% BSA at 37 °C.

Following the synthesis of the *galacto*-like configured allylic carbasugar chloride **1**–**4**, we tested their inhibitory activity against enzymes from two different GH families, an α-galactosidase (GH27) and a β-galactosidase (GH35). Shown in Figure 2 are the results from typical kinetic experiments for the covalent labeling of Human α-galactosidase (GalA) as a function of inhibitor concentration (other kinetic plots for covalent labeling are given in the Supplementary Information; Figure S1: Human α-galactosidase by **3**, and Figures S2–S4: *A. oryzae* β-galactosidase by **1**–**3**).

**Figure 3:**
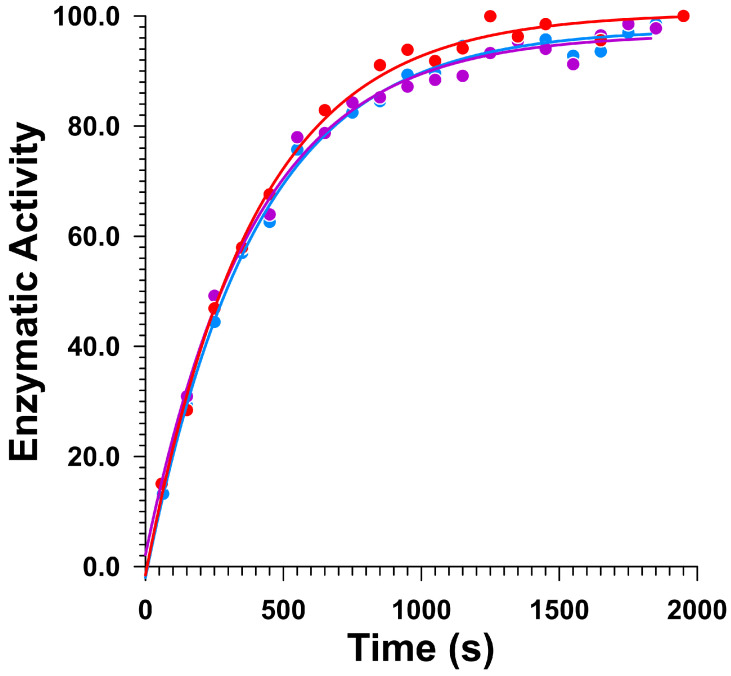
The plot of enzyme activity recovery for the GH35 β-galactosidase from *A. oryzae* following covalent inhibition by **1**, **2**, and **3**. The return of enzymatic activity after covalent inhibition by cyclohexenyl-based carbasugars **1** (blue circles), **2** (purple circles), and **3** (red circles) occurs in a first-order process, and all lines are the least-squares fit to such a standard exponential rate law. All experiments were run in 50 mM sodium acetate buffer, pH 4.5, and 0.1% BSA at T = 37 °C.

In summary, all inhibitors that covalently label either Human α-galactosidase or *Aspergillus oryzae* β-galactosidase display linear second-order kinetics where the slopes of the graphs give *k*_inact_/*K*_i_ (Table 1 and Table 2). That is, no saturation binding is noted even up to inhibitor concentrations of 8 mM, i.e., *K*_i_ » 8 mM. As was the case for the *gluco*-configured cyclohexenyl carbasugars reported previously [23], the half-lives of the covalent intermediates, formed by the pseudogalactosylation of Human α-galactosidase and *A. oryzae* β-galactosidase by **1**–**3**, are less than one hour. As a result, reactivation kinetics were monitored by diluting a quantity of inhibited enzyme into a buffered solution containing substrate. Shown in Figure 3 are the measured gradients (time intervals of 100 s) from the absorbance versus time plot of the reactivation for GH35 *A. oryzae* β-galactosidase with **1**–**3**. The kinetic plot for the reactivation of Human α-galactosidase is given in Figure S5.

The tabulated kinetic parameters for enzyme covalent labeling and the subsequent reactivation event are given in Table 1 and Table 2.

### 2.3. Inhibitory Mechanism Discussion

#### 2.3.1. Covalent Inhibition and Reactivation of Human α-Galactosidase

Both the *galacto*-configured carbasugar (**2**) in which the leaving chloride ion occupies the position of the C6 hydroxymethyl group of natural substrates and the carbasugar that is missing the C6 hydroxymethyl group (**3**) are covalent inhibitors of Human α-galactosidase. Of note, the allylic carbasugar in which the endocyclic double bond is located between C5a and C1, positions that are occupied by the anomeric carbon and the ring oxygen atoms in natural substrates, is more active. That is, in a manner reminiscent of the glucocarbasugars [23], the more active allylic carbasugar chloride is the one that possesses a ground state ^4^*H*_3_ conformation, a structure that resembles that proposed for the formation of the glycopyranosylium ion-like TS for the hydrolysis of substrates (Figure 1a) [5,6,26]. Unsurprisingly, the β-carbasugar (**1**) and the l-carbasugar (**4**) display no measurable covalent inhibition at 5 mM.

**Figure 4:**
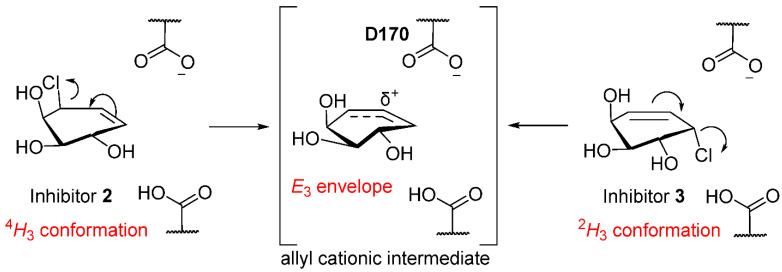
The proposed formation of an allylic cation in the active site of GH27 α-galactosidase from the reactions of covalent inhibitors **2** and **3**, which react from ^4^*H*_3_ and ^2^*H*_3_ conformations, respectively. The allylic cation intermediate (*E*_3_ conformation) is subsequently trapped by the active site nucleophile (D170) to give a β-galactosyl–enzyme intermediate.

The half-lives for hydrolysis of the galactocarbasugar-derived intermediates formed by **2** and **3** with Human α-galactosidase are identical, within experimental error, an observation that is consistent with the formation of the same intermediate containing a covalent bond between the pseudoanomeric carbon and the enzymatic nucleophile (D170) [23]. We conclude, based on the more rapid inactivation by **2** and the identical pseudodegalactosylation rate constants [23], that pseudogalactosylation with **2** and **3** occurs via dissociative processes (S_N_1′ and S_N_1, respectively) that give rise to a common enzyme-bound allylic cationic intermediate (Figure 4). This cationic intermediate is then trapped by the enzymatic nucleophile to give the same allylic carbasugar–enzyme complex, which then undergoes enzyme-catalyzed hydrolysis (Figure S6, Supplementary Information).

#### 2.3.2. Covalent Inhibition and Reactivation of *A. Oryzae* β-Galactosidase (LacA)

**Figure 5:**
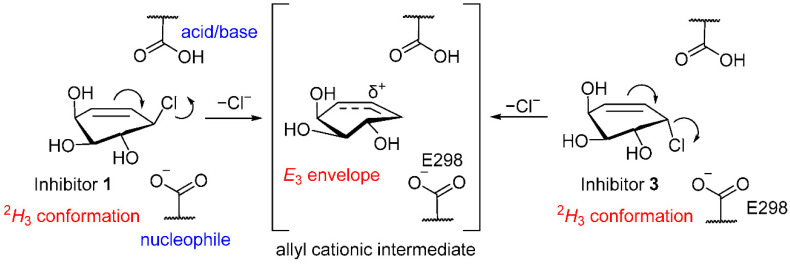
The proposed formation of an allylic cation in the active site of GH35 β-galactosidase from the reactions of epimeric covalent inhibitors **1** and **3**, which react from ^2^*H*_3_ conformations. The allylic cation intermediate (*E*_3_ conformation) is subsequently trapped by the active site nucleophile (E298) to give an α-carbasugar–enzyme intermediate.

Once again, the inhibitor (2) that has a ground state conformation (^4^*H*_3_) containing a C5 chloride leaving group has a second-order rate constant (*k*_inact_/*K*_i_) for covalent inhibition greater than that of the natural substrate mimic 1. That is, the more active inhibitor resembles the TS conformation for glycosylation that occurs during enzyme-catalyzed substrate turnover. Surprisingly, the α-galactocarbasugar chloride (3) also covalently inhibits the β-galactosidase from *A. oryzae* with a rate constant that is only about 6-fold less than that of the β-galactocarbasugar 1, while the C5 epimeric chloride (4) again shows no activity. Given that the active site nucleophile (E298) [27] is located on the same side as the chloride leaving group, it is clear that participation at the pseudoglycosylation TS is impossible. Moreover, the ratio of second-order rate constants (*k*_inact_/*K*_i_ ≈ 6.3) for covalent labeling by 3 and 1 is inconsistent with any nucleophilic assistance by E298 occurring during the reactions of 3 (Figure 5). We therefore conclude that these carbasugars react within a flexible GH35 enzyme’s active site [28] via dissociative S_N_1 and S_N_1′ mechanisms that proceed through a common, albeit short-lived, enzyme-bound allylic cation [23].

Once more, the half-lives for the hydrolysis of the galactocarbasugar-derived covalent intermediates formed by **1**, **2,** and **3** with the β-galactosidase from *A. oryzae* are identical, and we conclude that all three inhibitors form the same covalent intermediate that has a C-O bond between the pseudoanomeric carbon and the enzymatic nucleophile (E298) [27].

In summary, we have shown that cyclohexenyl-based carbasugars with a good chloride leaving group covalently label both α-galactosidase and β-galactosidase enzymes through the formation of allylic cation intermediates within the active site. The *A. oryzae* β-galactosidase is covalently labeled by three isomeric inhibitors (**1**–**3**), and importantly, these reactions involve neither acid catalysis (chloride ion departure) nor nucleophilic assistance but are catalyzed by the electrostatic environment within the active site. Finally, we suggest that such carbasugar chlorides should, with the correct hydroxyl group stereochemistry, be a general class of reversible inhibitors for typical retaining GHs.

#### 2.3.3. Conformational Itinerary of Carbasugar Inhibitors during Covalent Inhibition and Reactivation of Human α-Galactosidase (GalA) and A. Oryzae β-Galactosidase (LacA)

The proposed conformational itinerary, based on X-ray diffraction studies, for the Human α-galactosidase hydrolysis of substrates involves the Michaelis complex being a ^4^*C*_1_ chair and the covalent intermediate being a ^1^*S*_3_ skew boat, which is formed by traversing a ^4^*H*_3_^‡^ half-chair transition state [26]. We propose that carbasugars **2** and **3** react with GalA to give the covalent intermediate that is either in a ^2^*H*_3_ half-chair or a ^2^*E* envelope [23], with the conformational itinerary for **2** being ^4^*H*_3_ → *E*_3_^‡^ → ^2^*H*_3_/^2^*E*, while the less reactive **3** requires the formation of the *E*_3_ transition state from a ^2^*H*_3_ ground state (Figure S7, Supplementary Information). For the *A. oryzae* β-galactosidase, the conformational itineraries for covalent inhibition will likely be the same for **2** ^4^*H*_3_ → *E*_3_^‡^ → ^2^*H*_3_/^2^*E* and ^2^*H*_3_ → *E*_3_^‡^ → ^2^*H*_3_/^2^*E* for the reactions of both **1** and **3**.

## 3. Materials and Methods

### 3.1. Chemistry

We acquired nuclear magnetic resonance (NMR) spectra on one of a Bruker Avance 600 using either a QNP or a TCI cryoprobe (600 MHz), a Bruker 500 (500 MHz), or a Bruker 400 (400 MHz) at room temperature. The following fully deuterated solvents, acetone, acetonitrile, chloroform, methanol, deuterium oxide, and anhydrous dimethylsulfoxide, were purchased and used without further purification. Spectral data were reported as follows: chemical shift (multiplicity [singlet (s), broad singlet (bs), doublet (d), triplet (t), quartet (q), multiplet (m), doublet of doublets (dd), doublet of doublet of doublets (ddd), doublet of triplets (dt)], coupling constant, integration). Chemical shifts (δ) were listed in ppm downfield from TMS based on reported chemical shift of the ^1^H resonances that were referenced to solvent residual peaks [29]. Coupling constants were reported in Hz. All ^1^H and ^13^C NMR resonances were assigned using information from ^1^H-^1^H COSY, ^1^H-^13^C Heteronuclear Single Quantum Coherence, and Heteronuclear Multiple Bond Correlation experiments. Routine ^13^C NMR spectra were acquired in proton decoupling mode on either a 500 or 600 MHz spectrometer. ^13^C resonances were reported in ppm relative to solvent residual peaks [29]. We used an Agilent 6210 TOF LC/MS (Agilent Technologies, Santa Clara, CA, USA) using ESI-MS to acquire high-resolution mass spectra. Any reaction that required strictly dry conditions was run in oven-dried glassware under an atmosphere of argon. We used Merck silica gel 60 F254 plates (Merck KGaA, Darmstadt, Germany) for TLC analyses, using either UV light and/or a phosphomolybdic acid stain (5% solution in EtOH) for visualization. Silica column chromatography involved either a Combiflash nextgen plus (Teledyne, Thousand Oaks, CA, USA) or Avanco or Merck silica gel 60 (230–400 mesh). All starting materials and solvents, purchased from Sigma Aldrich, Alfa Aesar, TCI America, Combi-Blocks, Arcos, EMD, Anachemia, Caledon, Fisher, or ACP, were analytical grade or better and used without further purification unless noted otherwise. Anhydrous tetrahydrofuran (THF, dried over Na) was freshly distilled, and dry dichloromethane was distilled (CaH_2_). Cold temperatures were maintained by use of the following conditions: at 0 °C, ice-water bath was used; for reactions/work-up run-in water (H_2_O), deionized water was used unless stated otherwise. Room temperature (rt) is defined as 23–25 °C. Abbreviations used were as follows: – AcOH: acetic acid; CHCl_3_: chloroform; DCM or CH_2_Cl_2_: dichloromethane; DIAD: diisopropyl azodicarboxylate; DMF: *N*,*N*-dimethylformamide; EtOAc or EA: ethyl acetate; LiCl: lithium chloride; MeOH: methanol or methyl alcohol; Ms-Cl: methanesulfonyl chloride; NH_4_Cl: ammonium chloride; NaHCO_3_: sodium bicarbonate; Na_2_SO_4_: sodium sulfate; PMB-Cl: *p*-methoxybenzyl chloride; PPTS: pyridinium *p*-toluenesulfonate; TBA-Cl: *n*-tetrabutylammonium chloride; TBAF: *n*-tetrabutylammonium fluoride; TFA: trifluoroacetic acid; THF: tetrahydrofuran; TPP: triphenylphosphine; TBS-Cl: *tert*-butyl dimethyl silyl chloride; TMSOTf: trimethylsilyl trifluoromethanesulfonate.

*4-(t-Butyldimethylsilyl)-2,3-O-isopropylidene-1-(4-methoxybenzyl)-5,5a-didehydro-5a-carba-*α*-*d*-lyxo-hexopyranose* (**6**): We added solutions of **5** (1.00 g, 3.33 mmol, 1.0 equiv.) in anhydrous DMF (6.6 mL, 2 mL·mmol^−1^) dropwise at 0 °C to a stirred suspension of sodium hydride (0.200 g (60% *w*/*w*), 4.99 mmol, 1.5 equiv.) in anhydrous *N*,*N*-DMF (10 mL, 2 mL·mmol^−1^), and we stirred this reaction mixture for 1h. Then, we added PMB-Cl (0.5 mL, 3.66 mmol, 1.1 equiv.) dropwise at 0 °C, and we allowed the resultant mixture to stir for 1h. We monitored the reaction using TLC analysis, and when it was complete, it was poured onto crushed ice (20 g). This mixture was then extracted with ethyl acetate (30 mL × 3), and the organic layer was washed with brine (25 mL), dried over anhydrous sodium sulfate, and filtered through a stem funnel using cotton. After the solution was concentrated under reduced pressure, we obtained the crude product that we purified by column chromatography using 5% to 25% ethyl acetate in hexanes gradient to give compound **6** (0.991 g, 2.36 mmol, 71%) as a clear oil (TLC, EtOAc/hexane 20:80 *v*/*v*, *R*_f_ = 0.7).

**6**: ^1^H NMR (CDCl_3_, 600 MHz) δ 7.32 (d, *J* = 8.6, 2H, Ar–*H*), 6.88 (d, *J* = 8.6, 2H, Ar–*H*), 5.69 (ddd, *J* = 10.1, 2.9, 1.9, 1H, –Olefin-C*H*), 5.63 (ddd, *J* = 10.1, 2.9, 1.9, 1H, –Olefin-C*H*), 4.73 (d, *J* = 11.5, 1H, –OCH–*H*), 4.61 (d, *J* = 11.6, 1H, –OCH–*H*), 4.20 (dd, *J* = 8.1, 5.4, 1H, –OC*H*), 4.08 (dt, *J* = 6.6, 2.3, 1H, –OC*H*), 3.99 (dd, *J* = 8.1, 6.0, 1H, –OC*H*), 3.94 (dq, *J* = 5.0, 2.3, 1H, –OC*H*), 3.80 (s, 3H, Ar–OC*H_3_*), 1.46 (s, 3H, –C*H_3_*), 1.36 (s, 3H, –C*H_3_*), 0.91 (s, 9H, –C(C*H_3_*)_3_), 0.11 (s, 3H, –Si-C*H_3_*), 0.10 (s, 3H, –Si-C*H_3_*); ^13^C NMR (CDCl_3_, 151 MHz) δ 159.29, 132.65, 130.45, 129.60 (2C), 128.25, 113.88 (2C), 109.07, 79.91, 78.70, 77.45, 72.17, 71.36, 55.43, 27.43, 26.00 (3C), 25.10, 18.37, -4.34, -4.82; HRMS (ESI^+^) m/z calcd. For C_23_H_36_O_5_SiNa (M + Na)^+^ 443.2230. Found 443.2238.

*2,3-O-Isopropylidene-1-(4-methoxybenzyl)-5,5a-didehydro-5a-carba-*α*-*d*-lyxo-hexopyranose* (**7**): We made an ice-cold solution of **6** (0.970 g, 2.31 mmol, 1.0 equiv.) in dry THF (11.5 mL, 5 mL·mmol^−1^) and then added solution TBAF in THF (1 M, 2.54 mL, 2.54 mmol, 1.1 equivalent). We followed loss of the silyl ether with TLC analysis, and we quenched the reaction after 4 h. Subsequently, we diluted the resultant mixture with ethyl acetate (50 mL) and washed it with cold saturated sodium bicarbonate (2 × 25 mL). We then washed the separated organic layer with brine (25 mL), and we dried it over anhydrous sodium sulfate and filtered it through a stem funnel using cotton. We concentrated the resultant organic phase under reduced pressure to obtain a crude product that we purified by column chromatography using 5% to 25% ethyl acetate in hexanes gradient to provide us with compound **7** (0.612 g, 2.31 mmol, 86%) as a clear oil (TLC, EtOAc/hexane 20:80 *v*/*v*, *R*_f_ = 0.4).

**7**: ^1^H NMR (CDCl_3_, 600 MHz) δ 7.28 (d, *J* = 8.6, 2H, Ar–*H*), 6.88 (d, *J* = 8.6, 2H, Ar–*H*), 6.01 (ddd, *J* = 9.9, 3.5, 1.6, 1H, –Olefin-C*H*), 5.95 (ddd, *J* = 9.9, 3.4, 1.6, 1H, –Olefin-C*H*), 4.64 (d, *J* = 11.4, 1H, –OCH_2_–*H*), 4.59 (d, *J* = 11.4, 1H, –OCH_2_–*H*), 4.41 (dd, *J* = 7.7, 4.0, 1H, –OC*H*), 4.22 (dd, *J* = 7.7, 5.0, 1H, –OC*H*), 4.12 (ddq, *J* = 6.9, 3.5, 1.7, 1H, –OC*H*), 4.01 (tt, *J* = 3.2, 1.4, 1H, –OC*H*), 3.80 (s, 3H, Ar–OC*H_3_*), 2.61 (d, *J* = 7.0, 1H, CH–O*H*), 1.43 (s, 3H, –C*H_3_*), 1.36–1.36 (m, 3H, –C*H_3_*); ^13^C NMR (CDCl_3_, 151 MHz) δ 159.47, 132.58, 129.88, 129.85, 129.72 (2C), 113.99 (2C), 109.16, 79.08, 77.73, 75.06, 71.28, 69.10, 55.44, 26.93, 24.77; HRMS (ESI^+^) m/z calcd. For C_17_H_22_O_5_Na (M + Na)^+^ 329.1365. Found 329.1369.

*2,3-O-Isopropylidene-1-(4-methoxybenzyl)-4-(methanesulfonate)-5,5a-didehydro-5a-carba-*α*-*d*-lyxo-hexopyranose* (**8**): We added triethylamine (0.81 mL, 5.88 mmol, 3.0 equiv.) to a stirred solution of **7** (0.600 g, 1.96 mmol) in anhydrous DCM (9.8 mL, 5 mL·mmol^−1^) that was maintained at 0 °C. We then added MsCl (0.23 mL, 2.94 mmol, 1.5 equiv.) and continued the stirring at rt for a further 14 h. At this time, TLC analysis (EtOAc/hexane 30:70 *v*/*v*) showed no remaining starting material; we diluted the mixture with CH_2_Cl_2_ (50 mL), and we extracted it with cold saturated sodium bicarbonate (2 × 25 mL) and subsequently with brine (25 mL). We then dried the organic layer (Na_2_SO_4_) and filtered it using a stem funnel containing cotton wool, and then, finally, we evaporated the solvent using a vacuum to obtain a crude product that we purified by chromatography (EtOAc/hexanes, 5% to 25% gradient) to provide compound **8** (0.68 g, 1.96 mmol, 90%) as a clear oil (TLC, EtOAc/hexane 30:70 *v*/*v*, *R*_f_ = 0.5).

**8**: ^1^H NMR (CDCl_3_, 600 MHz) δ 7.30 (d, *J* = 8.6, 2H, Ar–*H*), 6.89 (d, *J* = 8.6, 2H, Ar–*H*), 5.88 (dt, *J* = 10.3, 2.5, 1H, –Olefin-C*H*), 5.80 (ddd, *J* = 10.3, 2.8, 2.0, 1H, –Olefin–C*H*), 4.94 (dq, *J* = 6.8, 2.4, 1H, –OC*H*), 4.71 (d, *J* = 11.4, 1H, –OCH_2_–*H*), 4.60 (d, *J* = 11.4, 1H, –OCH_2_–*H*), 4.32 (dd, *J* = 8.0, 4.4, 1H, –OC*H*), 4.22 (dd, *J* = 8.0, 6.5, 1H, –OC*H*), 4.01 (dq, *J* = 4.7, 2.4, 1H, –OC*H*), 3.81 (s, 3H, Ar–OC*H_3_*), 3.15 (s, 3H, –OSO_2_–C*H_3_*), 1.51 (s, 3H, –C*H_3_*), 1.39 (s, 3H, –C*H_3_*); ^13^C NMR (CDCl_3_, 151 MHz) δ 159.49, 131.19, 129.73 (2C), 126.43, 113.99 (2C), 110.01, 80.74, 77.90, 76.31, 76.09, 71.64, 55.44, 38.82, 27.25, 25.11; HRMS (ESI^+^) m/z calcd. For C_18_H_24_O_7_SNa (M + Na)^+^ 407.1140. Found 407.1145.

*Synthesis of carbasugars from mesylates* (**9a–9d**): After we made a solution of **8** (0.500 g, 1.30 mmol) in anhydrous DMF (6.5 mL, 5 mL·mmol^−1^), we added lithium chloride (0.551 g, 13.0 mmol, 10.0 equiv.) and then tetrabutylammonium chloride (0.361 g, 1.30 mmol, 1.0 equiv.). We continued stirring the resultant mixture until TLC analysis showed complete formation of product, a process that took 4 h. We then concentrated this mixture under reduced pressure to provide us with a crude product that we purified by chromatography (EtOAc/hexanes, 20% to 50% gradient), and we were afforded four compounds (TLC, EtOAc/hexane 30:70 *v*/*v*, *R*_f_ = 0.8 for **9a**, 0.7 for **9b** and **9d** inseparable mixture, 0.6 for **9c**). The compounds labeled as **9a** and **9c** were isolated as pure products. Compounds **9b** and **9d** were isolated as a mixture in a 35:65 ratio.

**9a**: ^1^H NMR (CDCl_3_, 600 MHz) δ 7.31 (d, *J* = 8.6, 2H), 6.89 (d, *J* = 8.6, 2H), 5.86 (dt, *J* = 10.2, 2.3, 1H), 5.80 (dt, *J* = 10.1, 2.4, 1H), 4.71 (d, *J* = 11.5, 1H), 4.60 (d, *J* = 11.5, 1H), 4.34 (dd, *J* = 6.7, 4.4, 1H), 4.30–4.25 (m, 2H), 4.00 (dt, *J* = 3.6, 2.0, 1H), 3.81 (s, 3H), 1.49 (s, 3H), 1.40 (s, 3H); ^13^C NMR (CDCl_3_, 151 MHz) δ 159.43, 130.51, 129.98, 129.70 (2C), 128.94, 113.95 (2C), 109.52, 79.52, 78.80, 75.94, 71.42, 57.12, 55.44, 27.38, 25.05; HRMS (ESI^+^) m/z calcd. For C_17_H_21_O_4_ClNa (M + Na)^+^ 347.1026. Found 347.1029.

**9c**: ^1^H NMR (CDCl_3_, 600 MHz) δ 7.38 (d, *J* = 8.6, 2H), 6.88 (d, *J* = 8.6, 2H), 5.95–5.84 (m, 2H), 4.88 (d, *J* = 10.6, 1H), 4.75 (d, *J* = 10.6, 1H), 4.62 (ddd, *J* = 6.3, 2.9, 1.4, 1H), 4.41 (dd, *J* = 8.3, 1.4, 0H), 4.20 (dd, *J* = 8.3, 6.2, 1H), 3.66 (t, *J* = 8.3, 1H), 1.52 (s, 3H), 1.41 (s, 3H); ^13^C NMR (CDCl_3_, 151 MHz) δ 159.45, 132.51, 130.39, 130.03 (2C), 124.61, 113.86 (2C), 110.53, 81.53, 78.20, 74.80, 72.42, 57.97, 55.44, 28.27, 26.17; HRMS (ESI^+^) m/z calcd. For C_17_H_21_O_4_ClNa (M + Na)^+^ 347.1026. Found 347.1029.

Inseparable mixture of 2,3-O-isopropylidene-4-(4-methoxybenzyl)-5,5a-didehydro-5a-carba-α-l-lyxo-hexopyranosyl chloride and 3,4-O-isopropylidene-2-(4-methoxybenzyl)-5,5a-didehydro-5a-carba-α-l-arabino-hexopyranosyl chloride (**9b**) and (**9d**): ^1^H NMR (CDCl_3_, 400 MHz) δ 7.37–7.26 (m, 2H and 3.3H), 6.94–6.82 (m, 2H and 3.3H), 6.12–5.92 (m, 2H and 3.3H), 4.79–4.65 (m, 1H and 3.3H), 4.63–4.52 (m, 3.3H), 4.48 (m, 3.3H and 2H), 4.32–4.24 (m, 1.7H), 3.80 (s, 5.1H and 3H), 3.69 (dd, J = 8.0, 3.7, 1H), 1.52 (s, 5.1H), 1.42 (s, 3H), 1.40–1.36 (s, 5.1H and 3H); HRMS (ESI^+^) m/z calcd. For C_17_H_21_O_4_ClNa (M + Na)^+^ 347.1026. Found 347.1029.

*5,5a-didehydro-5a-carba-β-l-arabino-hexopyranosyl chloride* (**1**): To a rt stirred solution of **9c** (0.050 g, 0.154 mmol) in anhydrous DCM (1.5 mL, 10 mL·mmol^−1^), we added TFA (0.12 mL, 1.539 mmol, 10 equiv.). We noted that after 3 h, the reaction was complete (TLC analysis), and we then concentrated it under reduced pressure. We purified the resultant crude product by chromatography (MeOH/DCM, 5% to 15% gradient) to afford us **1** (17.9 mg, 0.109 mmol, 71%) as a white solid (TLC, MeOH/DCM 10:90 *v*/*v*, *R*_f_ = 0.4).

**1**: ^1^H NMR (CD_3_OD, 600 MHz) δ 5.87 (ddd, *J* = 10.0, 5.3, 2.1, 1H, *H5*), 5.78 (dd, *J* = 10.0, 2.2, 1H, *H5a*), 4.34 (dtd, *J* = 8.0, 2.2, 0.9, 1H, *H1*), 4.18 (t, *J* = 4.7, 1H, *H4*), 3.85 (dd, *J* = 10.4, 8.1, 1H, *H2*), 3.45 (dd, *J* = 10.4, 4.1, 1H, *H3*); ^13^C NMR (CD_3_OD, 151 MHz) δ 131.86 (*C5a*), 129.36 (*C5*), 74.40 (*C2*), 73.29 (*C3*), 67.73 (*C4*), 62.97 (*C1*); HRMS (ESI^+^) m/z calcd. For C6H9O3ClNa (M + Na)^+^ 187.0138. Found 187.0141.

*5,5a-Didehydro-5a-carba-β-l-lyxo-hexopyranosyl chloride* (**2**): We used an identical protocol to that for **1** and obtained **2** (11.4 mg, 0.069 mmol, 75%) as a white solid (TLC, MeOH/DCM 10:90 *v*/*v*, *R*_f_ = 0.5) from the following quantities: **9a** (0.030 g, 0.092 mmol) DCM (1.0 mL, 10 mL·mmol^−1^), and trifluoroacetic acid (0.09 mL, 0.924 mmol, 10 equiv.).

**2**: ^1^H NMR (CD_3_OD, 600 MHz) δ 5.78–5.73 (m, 2H, *H5*, *and H5a*), 4.48 (dq, *J* = 4.9, 1.1, 1H, *H1*), 4.19 (dd, *J* = 6.1, 1.8, 1H, *H4*), 4.01 (dd, *J* = 4.9, 2.4, 1H, *H2*), 3.87 (dd, *J* = 6.1, 2.4, 1H, *H3*); ^13^C NMR (CD_3_OD, 151 MHz) δ 132.11 (*C5a*), 128.34 (*C5*), 74.56 (*C2*), 73.14 (*C3*), 70.15 (*C4*), 59.15 (*C1*); HRMS (ESI^+^) m/z calcd. For C_6_H_9_O_3_ClNa (M + Na)^+^ 187.0138. Found 187.0142.

*5,5a-Didehydro-5a-carba-*α*-l-lyxo-hexopyranosyl chloride and 5,5a-didehydro-5a-carba-*α*-l-arabino-hexopyranosyl chloride* (**3**) and (**4**): We deprotected the mixture of **9b** and **9d** (0.060 g, 0.185 mmol) using a solution of TFA (0.143 mL, 1.847 mmol, 10 equiv.) in anhydrous CH_2_Cl_2_ (1.9 mL, 10 mL·mmol^−1^) at 25 °C with stirring for 3 h. We note that using an identical chromatographic solvent, we separated the two inhibitors and obtained **3** (7.5 mg, 0.046 mmol, 25%; TLC, MeOH/DCM 10:90 *v*/*v*, *R*_f_ = 0.5) and **4** (13.8 mg, 0.0838 mmol, 45%, TLC, MeOH/DCM 10:90 *v*/*v*, *R*_f_ = 0.4) as colorless solids.

**3**: ^1^H NMR (CD_3_OD, 600 MHz) δ 5.85 (ddd, *J* = 9.9, 4.1, 0.7, 1H, *H5a*), 5.81 (dd, *J* = 9.9, 4.3, 1H, *H5*), 4.73 (dd, *J* = 4.5, 3.4, 1H, *H1*), 4.26 (t, *J* = 4.1, 1H, *H4*), 4.05 (dd, *J* = 8.8, 4.0, 1H, *H2*), 3.91 (dd, *J* = 8.8, 4.2, 1H, *H3*); ^13^C NMR (CD_3_OD, 151 MHz) δ 131.28 (*C5*), 129.51 (*C5a*), 70.71 (*C2*), 69.75 (*C3*), 67.56 (*C4*), 60.28 (*C1*); HRMS (ESI^+^) m/z calcd. For C_6_H_9_O_3_ClNa (M + Na)^+^ 187.0138. Found 187.0140.

**4**: ^1^H NMR (CD_3_OD, 600 MHz) δ 5.68 (dt, *J* = 10.2, 2.3, 1H, *H5a*), 5.56 (dq, *J* = 10.3, 2.0, 1H *H5*), 4.81 (ddd, *J* = 5.5, 3.2, 2.3, 1H, *H1*), 4.30 (dq, *J* = 7.6, 2.4, 1H, *H4*), 4.07 (dt, *J* = 3.6, 1.9, 1H, *H2*), 3.54 (dd, *J* = 8.0, 2.1, 1H, *H3*); ^13^C NMR (CD_3_OD, 151 MHz) δ 132.21 (*C5a*), 127.91 (*C5*), 76.25 (*C3*), 74.32 (*C2*), 70.01 (*C4*), 60.30 (*C1*); HRMS (ESI^+^) m/z calcd. For C_6_H_9_O_3_ClNa (M + Na)^+^ 187.0138. Found 187.0141.

### 3.2. Measurement of Rate Constants for Galactosidase Covalent Inhibition

Recombinant Human α-galactosidase (GalA) was purchased from Biotechne, and *Aspergillus oryzae* β-galactosidase was purchased from Sigma. We calculated the various rate constants for covalent inhibition of these two glycosidases by compounds **1** to **4** by use of either a classical jump dilution assay [30] or a continuous assay protocol [31].

First, we incubated the enzyme using at least five different inhibitor concentrations in the appropriate buffer. We then ‘jump-diluted’ the inactivation mixture by adding 4 µL aliquots of the inhibition mixture to a pre-equilibrated 196 µL buffered solution containing substrate (see assay conditions section). We monitored the enzymatic activity using either UV-vis (Cary300, Agilent Technologies, Santa Clara, CA, United States) or fluorescence (Cary Eclipse). All rate constants for inhibition (*k*_obs_) were calculated using a standard nonlinear least squares fit of enzyme activity (%) versus time data to a first-order equation. We then used a standard Michaelis–Menten expression to evaluated *k*_inact_ and *k*_inact/_*K*_i_ for covalent inhibition using a standard nonlinear least squares fitting routine. However, in cases where we noted insufficient saturation of inhibitor, we used a standard linear fit of the *k*_obs_ versus covalent inhibitor concentration to obtain the inhibition rate constant *k*_inact_/*K*_i_.

Second, we also used a continuous assay protocol in which we added the enzyme to a pre-equilibrated buffered mixture containing substrate and inhibitor. We monitored the reaction continuously until we noted that the rate of fluorophore (or chromophore) became linear following the initial first-order process. We repeated these experiments for at least five other inhibitor concentrations. We calculated the apparent rate constant (*k*_app_) at every inhibitor concentration by fitting the progress curves to equation 1 (for UV-Vis or a similar equation for fluorescence data), where *V*_i_ is the initial velocity at t = 0 and *V*_f_ is the velocity at t = ∞ [31].

(1) $$Abs=\nu_{f}t+\left(\nu_{i}-\frac{\nu_{f}}{k_{app}}\right)\left(1-e^{\left(-k_{app}t\right)}\right)$$

The *k*_app_ values versus inhibitor concentrations were then fit to a modified Michaelis–Menten expression for competitive inhibition (equation 2), which gives the second-order inhibition parameter *k*_inact_/*K*_i_ only [31].

(2) $$k_{app}=k_{react}+\frac{k_{inact}}{K_{i}}\left(\frac{\left[I\right]}{1+\left[S\right]/K_{m}}\right)$$

The release of 4-nitrophenol was monitored at 400 nm, while the release of 4-methylumbelliferone was monitored at 365 nm excitation and 445 emission wavelengths. All rate constants were determined by fitting experimental data on the computer program GraphPad Prism 10.0 (GraphPad Software, Boston, MA, USA).

### 3.3. Measurement of Rate Constants for Galactosidase Reactivation

We monitored a concentrated solution of enzyme and inhibitor at 37 °C until the measured enzyme activity dropped to under 5% of that initially measured. We then added a small volume (typically 0.5 µL) to a pre-equilibrated reactivation buffer containing substrate (200 µL). We then continuously monitored the release of either the chromophore or the fluorophore [30]. We then calculated signal versus time gradient slope at uniform time intervals to give reaction rates. We fit these computed values to a standard first-order rate equation (Prism 10.0) to give us the first order reactivation rate constant (*k*_react_).

## Figures and Tables

**Table 1:** Calculated rate constants for the covalent inhibition of Human GH31 α-galactosidase (GalA). All experiments were performed in 50 mM sodium citrate buffer, pH 4.6, containing 50 mM NaCl and 0.1% BSA at 37 °C.

Compound	*k*_inact_/*K*_i_ (M^−1^ s^−1^)	*k*_react_ (s^−1^)	*t*_1/2_ (min)
**1**	NIO *^a^* at 5 mM	–	
**2**	0.25 ± 0.004	(5.1 ± 0.2) × 10^−4^	22.5 ± 0.7
**3**	0.18 ± 0.01	(4.9 ± 0.2) × 10^−4^	23.4 ± 0.9
**4**	NIO *^a^* at 5 mM	–	

*^a^* NIO = No inhibition observed.

**Table 2:** Calculated rate constants for the covalent inhibition of *A. oryzae* GH35 β-galactosidase (LacA): All experiments were performed in 50 mM sodium acetate buffer, pH 4.5, containing 0.1% BSA at 37 °C.

Compound	*k*_inact_/*K*_i_ (M^−1^ s^−1^)	*k*_react_ (s^−1^)	*t*_1/2_ (min)
**1**	17.0 ± 0.5	(2.5 ± 0.1) × 10^−3^	4.6 ± 0.2
**2**	47 ± 2	(2.6 ± 0.2) × 10^−3^	4.5 ± 0.3
**3**	2.7 ± 0.04	(2.6 ± 0.2) × 10^−3^	4.5 ± 0.3
**4**	NIO *^a^* at 1 mM	–	–

*^a^* NIO = No inhibition observed.

## Data Availability

Data are contained within the article and the Supplementary Information.

## Supplementary Materials

The following supporting information can be downloaded at https://www.mdpi.com/article/10.3390/molecules29204870/s1. Tables of kinetic data, plots of kinetic data, and ^1^H, ^13^C {^1^H}, COSY, HSQC, and HMBC NMR spectra for covalent inhibitors **1**–**4**.
